# SARC006: Phase II Trial of Chemotherapy in Sporadic and Neurofibromatosis Type 1 Associated Chemotherapy-Naive Malignant Peripheral Nerve Sheath Tumors

**DOI:** 10.1155/2017/8685638

**Published:** 2017-09-12

**Authors:** Christine S. Higham, Seth M. Steinberg, Eva Dombi, Arie Perry, Lee J. Helman, Scott M. Schuetze, Joseph A. Ludwig, Arthur Staddon, Mohammed M. Milhem, Daniel Rushing, Robin L. Jones, Michael Livingston, Stewart Goldman, Christopher Moertel, Lars Wagner, David Janhofer, Christina M. Annunziata, Denise Reinke, Lauren Long, David Viskochil, Larry Baker, Brigitte C. Widemann

**Affiliations:** ^1^Pediatric Oncology Branch, NCI, CCR, Bethesda, MD, USA; ^2^Biostatistics and Data Management Section, NCI, Bethesda, MD, USA; ^3^University of California, San Francisco, San Francisco, CA, USA; ^4^University of Michigan Comprehensive Cancer Center, Ann Arbor, MI, USA; ^5^MD Anderson Cancer Center, Houston, TX, USA; ^6^University of Pennsylvania, Abramson Cancer Center, Philadelphia, PA, USA; ^7^University of Iowa Hospital and Clinics, Iowa City, IA, USA; ^8^Indiana University, Indianapolis, IN, USA; ^9^University of Washington, Fred Hutchinson Cancer Research Center, Seattle, WA, USA; ^10^The Royal Marsden NHS Foundation Trust, Institute of Cancer Research, London, UK; ^11^Levine Cancer Center, Charlotte, NC, USA; ^12^Ann and Robert H. Lurie Children's Hospital of Chicago, Chicago, IL, USA; ^13^University of Minnesota Masonic Children's Hospital, Minneapolis, MN, USA; ^14^Cincinnati Children's Hospital Medical Center, Cincinnati, OH, USA; ^15^Women's Malignancies Branch, NCI, Bethesda, MD, USA; ^16^SARC, Ann Arbor, MI, USA; ^17^University of Utah, Salt Lake City, UT, USA

## Abstract

**Background:**

Worse chemotherapy response for neurofibromatosis type 1- (NF1-) associated compared to sporadic malignant peripheral nerve sheath tumors (MPNST) has been reported.

**Methods:**

We evaluated the objective response (OR) rate of patients with AJCC Stage III/IV chemotherapy-naive NF1 MPNST versus sporadic MPNST after 4 cycles of neoadjuvant chemotherapy, 2 cycles of ifosfamide/doxorubicin, and 2 cycles of ifosfamide/etoposide. A Simon optimal two-stage design was used (target response rate 40%).

**Results:**

34 NF1 (median age 33 years) and 14 sporadic (median age 40 years) MPNST patients enrolled. Five of 28 (17.9%) evaluable NF1 MPNST patients had a partial response (PR), as did 4 of 9 (44.4%) patients with sporadic MPNST. Stable disease (SD) was achieved in 22 NF1 and 4 sporadic MPNST patients. In both strata, results in the initial stages met criteria for expansion of enrollment. Only 1 additional PR was observed in the expanded NF1 stratum. Enrollment was slower than expected and the trial closed before full accrual.

**Conclusions:**

This trial was not powered to detect differences in response rates between NF1 and sporadic MPNST. While the OR rate was lower in NF1 compared to sporadic MPNST, qualitative responses were similar, and disease stabilization was achieved in most patients.

## 1. Introduction

Malignant peripheral nerve sheath tumors (MPNST) are soft tissue sarcomas associated with high risk of local recurrence, hematogenous metastasis, and high sarcoma-specific death rate [1–4]. They account for approximately 4% of all soft tissue sarcomas and occur mainly in adults, with only 10–20% occurring in patients less than 20 years old [5–8]. Half of MPNST arise in patients with neurofibromatosis type 1 (NF1), with a lifetime incidence of 15.8% in NF1 compared to an incidence of 0.001% in the general population [1, 9, 10]. In NF1, MPNST occurs at a younger age and the majority arise in preexisting plexiform neurofibromas (PNs) [11, 12].

Most patients present with a soft tissue mass that arises from a peripheral or major nerve and may have symptoms of pain, paresthesias, or weakness [13]. The diagnosis in patients with NF1 can be delayed because clinical indicators of malignancy such as mass and pain can also be features of benign PNs. Worse 5-year overall survival has been reported for NF1 MPNST (32%, 11%) compared to sporadic MPNST (55%, 44.7%), though, in recent years, studies have shown this difference is decreasing [3, 14, 15]. A French group recently reported that in patients with MPNST NF1 status was not negatively prognostic except in patients with recurrence or metastasis [16].

To date, complete surgical resection with wide negative margins is the only potentially curative treatment option for localized MPNST. Radiation treatment at high doses has been recommended for tumors ≥ 5 cm and in the presence of microscopic positive margins [17]. While the use of dose-intensive neoadjuvant chemotherapy is standard for patients with rhabdomyosarcoma and Ewing sarcoma, the survival benefits of this strategy are less established for other types of soft tissue sarcoma, though current clinical trials are underway [18–21]. Although several small phase II trials have reported that preoperative chemotherapy may increase the ability to successfully perform conservative surgery, the impact of this strategy on overall survival has not been well prospectively addressed [22, 23]. Doxorubicin and ifosfamide are considered the most active agents in treating soft tissue sarcomas, and etoposide has shown some increased response when combined with ifosfamide [24–27]. A retrospective analysis of EORTC trials found that doxorubicin-ifosfamide regimens are superior to other regimens and outcomes for MPNST are similar to those of other soft tissue sarcomas [28]. Because topoisomerase II*α (TOPO2A)* has been reported as the most overexpressed gene in MPNST compared to benign neurofibromas, targeting with a topoisomerase II inhibitor such as etoposide may be well suited for this particular type of sarcoma [29].

There is limited knowledge of the response rate of MPNST to standard chemotherapy agents used to treat pediatric and adult sarcomas. Two studies reported lower response to chemotherapy (partial response rates 8%–18% in NF1 MPNST versus 55%–60% in sporadic MPNST) and worse survival (5-year OS 11–32% versus 44–55%, resp.) in NF1-associated MPNST compared to sporadic MPNST. However, these studies were not designed prospectively and used a variety of chemotherapy agents on different protocols [3, 14].

The rationale for neoadjuvant therapy is severalfold, including (1) chemotherapy induced tumor shrinkage leading to easier and less morbid resection, (2) treatment of micrometastatic disease at diagnosis, and (3) response to chemotherapy can be assessed in individual patients allowing for tailoring of further treatment. The aim of this phase II trial was to prospectively determine the imaging response (WHO criteria) of AJCC Stage III/IV sporadic or NF1-associated MPNST after neoadjuvant chemotherapy with 2 cycles of ifosfamide and doxorubicin (IA), followed by 2 cycles of ifosfamide and etoposide (IE). Patients were stratified for NF1-associated versus sporadic MPNST to assess response in these groups separately.

## 2. Methods

### 2.1. Study Oversight

This multi-institutional trial was sponsored by the United States Department of Defense (DoD), developed by NCI investigators in collaboration with the Sarcoma Alliance for Research through Collaboration (SARC) and coordinated by SARC. The protocol was approved by the local institutional review board at each participating site and registered on ClinicalTrials.gov NCT00304083. All patients or their legal guardians provided written informed consent and assent (patients 13 through 17 years old).

### 2.2. Patient Eligibility

Children and adults (no upper or lower age limit) with measurable, high-grade (stage III per AJCC TNM staging system) or metastatic (stage IV) sporadic or NF1-associated MPNST not previously treated with chemotherapy for MPNST were eligible for the study. Organ function requirements included a normal serum creatinine for age or creatinine clearance > 60 mL/min/1.73 m^2^, alanine aminotransferase < 5x the upper limit of normal and bilirubin < 2.5x the upper limit of normal, absolute neutrophil count ≥ 1500/mcL, hemoglobin ≥ 9.0 g/dL, platelet count ≥ 100,000/mcL, and normal left ventricular ejection fraction on MUGA scan or echocardiogram.

### 2.3. Trial Design

#### 2.3.1. Treatment Regimen

**Figure 1:**
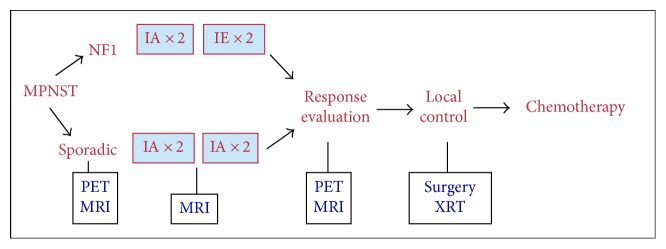
Trial design and treatment plan.

Patients were stratified for the presence of NF1-associated (stratum 1) or sporadic (stratum 2) MPNST. NF1 clinical findings were documented at enrollment using a standardized form. In each stratum, patients received 2 cycles (one cycle = 21 days) of doxorubicin and ifosfamide (IA) followed by 2 cycles of ifosfamide and etoposide (IE) (Figure 1). Mesna, peg-filgrastim or filgrastim, and dexrazoxane were given per institutional guidelines. Details regarding trial design, chemotherapy dosing, and supportive therapy are included in Supplementary Appendix Table S1 (see Supplementary Material available online at https://doi.org/10.1155/2017/8685638). If the start of the second or subsequent treatment cycles was delayed for >7 days by a nonhematologic toxicity that had not resolved to a level that would allow for initiation of the next treatment cycle, then the dose of agents that were judged to be responsible for the prolonged toxicity could be reduced by 25%.

Patients underwent local control (radiation and/or surgery), if feasible, after cycle 4, followed by 2 more cycles of IA and IE, for a planned 8 total chemotherapy cycles. The cumulative doxorubicin, ifosfamide, and etoposide doses were 300 mg/m^2^, 72,000 mg/m^2^, and 2000 mg/m^2^, respectively. Response was assessed using standard MRI/CT after cycles 2, 4, 6, and 8. Patients who underwent only surgery received 2 more cycles of IA followed by 2 more cycles of IE. Those who underwent radiation could receive IE during radiation followed by 2 more cycles of IA, as doxorubicin cannot be given concurrently with radiation. If patients developed disease progression after the first 2 cycles of IA, chemotherapy continued on protocol and they received 2 cycles of IE. Patients who experienced disease progression after 2 cycles each of IA and IE were permanently removed from the trial.

#### 2.3.2. Study Evaluations

Patients underwent regular safety evaluations: history, physical examinations, complete blood counts (CBC), electrolytes, liver function tests, and urinalysis prior to all cycles; CT chest and MRI of primary site prior to cycles 1, 3, 5, and 7; and, if possible, ^18^FDG-PET prior to cycles 1 and 5. CBC, electrolytes, and liver function tests were monitored at least weekly during treatment cycles (see Supplementary Appendix Table S2 for details).

#### 2.3.3. Evaluation of Toxicities

National Cancer Institute Common Terminology Criteria for Adverse Events, version 3, were used to grade adverse events.

#### 2.3.4. Response Criteria

WHO as opposed to RECIST criteria were used to determine responses due to the nonspherical shape of most MPNST [30, 31]. Responses were centrally reviewed at the NCI (ED). Patients were considered nonevaluable for response if they were removed from treatment for reasons other than disease progression or toxicity such as withdrawal of consent or noncompliance.

#### 2.3.5. Statistical Considerations

A Simon optimal two-stage design was used to evaluate response (complete and partial responses) to chemotherapy (WHO criteria) in order to rule out a 20% response rate (*p*_0_ = 0.20) and target a desirable goal of a 40% response rate (*p*_1_ = 0.40) separately within 2 strata: NF1-associated (stratum 1) and sporadic MPNST (stratum 2). Response evaluation after cycle 4 was used for determination of the primary trial endpoint. With alpha = 0.10 (probability of accepting a poor treatment) and beta = 0.10 (probability of rejecting a good treatment), in the first stage of each stratum, 17 patients were to be enrolled. If 0–3 patients responded, the regimen was to be considered inactive in that stratum and enrollment terminated. If ≥4 patients achieved partial response (PR) or complete response (CR), accrual would continue until a total of 37 patients were enrolled. The regimen would be considered active if ≥11 of 37 patients in the expanded stratum experienced a PR or CR; this would be consistent with a 40% response rate (the upper bound of a one-sided 90% CI would include 40%). With this design, the probability of early termination was 55% if the true response rate was only 20%. Secondary endpoints were to determine clinical phenotypes in NF1 patients with MPNST using a standardized evaluation form at trial entry, to perform a central, detailed, and standardized pathology evaluation (AP), compare response evaluation by RECIST versus WHO, and to evaluate the response of plexiform neurofibroma (if present) to neoadjuvant chemotherapy using magnetic resonance imaging (MRI) volumetric analysis.

#### 2.3.6. Central Pathology Review

The MPNST diagnosis was centrally confirmed. In addition, detailed analyses including determination of tumor margin, histologic variant, cellularity, number of mitoses, presence of tumor necrosis, grade, immunohistochemical features (including S100, p53, and MIB-1, a marker for cell proliferation), and fluorescence in situ hybridization (FISH) studies were performed for copy number alterations of 10 known tumor suppressor and oncogenes, including* TOPO2A* amplification on provided samples (see Table S3).

## 3. Results

### 3.1. Patient Characteristics

Between 9/2006 and 6/2012, 49 patients enrolled, 48 of whom were eligible. One patient was ineligible because on central pathology review the tumor was found to be a clear cell sarcoma. The characteristics of the 48 eligible patients are listed in Table 1. The majority of primary tumors were located in the trunk (*n* = 34, 24 NF1, 10 sporadic). Twenty-five patients had metastatic disease at the time of diagnosis (16 NF1, 9 sporadic). No patient had received prior chemotherapy or radiation therapy for their MPNST. Of the 26 patients with NF1 who had pathology available for review, 13 of the MPNST arose from a plexiform neurofibroma, 5 did not, and 8 were unknown. The majority of patients with NF1 had plexiform neurofibromas as well as multiple cutaneous and subcutaneous neurofibromas (Table 2).

### 3.2. Responses

Both initial stages met criteria for expansion of enrollment with 4/17 partial responses in NF1 and 5/9 partial responses in sporadic MPNST after cycle 4. The NF1 stratum went on to enroll 17 additional patients for a total of 34 patients and the sporadic stratum enrolled an additional 5 patients for a total of 14 patients. Enrollment on the trial was then closed due to slow enrollment and availability of competing clinical trials for MPNST. Overall, 37 of the 48 patients were evaluable for response (28 NF1, 9 sporadic MPNST). Patients were deemed nonevaluable for the following reasons; PI decision (*n* = 2), early local control (*n* = 3), patient withdrawal (*n* = 4), death during cycle 1 (*n* = 1) due to internal hemorrhage unrelated to therapy, and noncompliance (*n* = 1).

**Figure 2:**
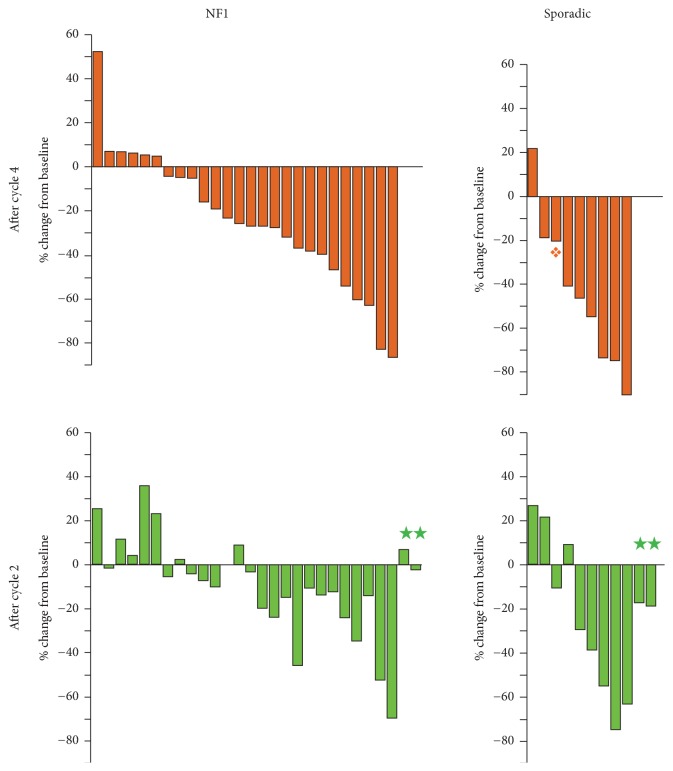
Waterfall plots showing change in 2-dimensional tumor measurements according to WHO criteria after 4 cycles (2 cycles ifosfamide and doxorubicin, IA, and 2 cycles of ifosfamide and etoposide, IE, upper panels) and 2 cycles (IA, lower panels). On all four panels patients are arranged by best response measured after cycle 4. ^★^Patients removed from treatment after cycle 2. ^❖^Target lesion stable, but progressive disease with new lesion.

In total, after cycle 4 evaluation, there were 9 patients with partial responses (5/28-17.9% NF1 MPNST, with exact 95% Confidence Interval (CI) 6.1–36.9%, and 4/9-44.4% sporadic MPNST, with exact 95% CI 13.7–78.8%) and 24 with stable disease (20 NF1 MPNST, 4 sporadic MPNST) and 4 patients with PD (3 NF1 MPNST, 1 sporadic MPNST) (see Table 1 and Figure 2, Supplementary Appendix Table S3). The objective response rate was thus lower in NF1 (17.9%) compared to sporadic (44.4%) MPNST. Of the 9 PR, 5 were initially observed after 2 cycles IA and 4 additional partial responses after completion of 2 cycles of IE. There were 4 patients with progressive disease after IA, one of whom had regression of tumor to SD after 2 cycles of IE. In addition to the patient with regression to SD at the end of cycle 4, 5 patients (4 NF1, 1 sporadic) had decrease in their MPNST after cycle 4 IE following initial growth from baseline after 2 cycles of IA.

In the NF1 stratum, the median number of treatment cycles was 5 (range 1–8); of the 26 patients who received ≥ 4 cycles, 12 completed all 8 cycles. For the sporadic stratum the median number of cycles was 4.5 (range 1–8) and of 10 patients who received ≥ 4 cycles, 6 completed all 8 cycles. The most frequent reasons for removal from treatment prior to completion of all 8 cycles were PI decision (8 NF1, 3 sporadic), disease progression (5 NF1, 1 sporadic), and patient withdrawal (4 NF1, 2 sporadic).

Of 22 patients in the NF1 stratum who remained on study after 4 cycles, 7 underwent surgery only, 5 had surgery and radiation, 4 received radiation only, and 6 had neither radiation nor surgery for local control. In the sporadic group, 7 patients remained on study after 4 cycles, 3 of whom underwent surgery only, 1 had surgery and radiation, 2 had radiation only, and 1 received neither radiation nor surgery for local control.

The secondary endpoint, to evaluate the response of plexiform neurofibroma (if present) to neoadjuvant chemotherapy using volumetric MRI analysis as a tool for response assessment, could not be evaluated. For most patients, MRI imaging of the MPNST and plexiform neurofibroma component was not sufficient to allow for volumetric analysis over time. Reasons for this included differences in imaging technique over time and incomplete coverage of the entire tumor (MPNST and preexisting plexiform neurofibroma).

For 33 patients who were evaluable for response after cycle 4, response evaluation using WHO and RECIST was performed. Response determination was in agreement with exception of two patients who had stable disease by WHO criteria (−39%, −46%) but a partial response by RECIST criteria (−30%, −31%), one patient who had progressive disease (52.6%) by WHO criteria and stable disease (18%) by RECIST criteria, and one patient with stable disease (−28%) by RECIST criteria but partial response (−54%) by WHO criteria.

### 3.3. Toxicity

Eight patients had dose reductions of chemotherapy with three of those being for neurotoxicity associated with ifosfamide. Serious adverse events with possible, probable, or definite relationship were reported for 9 patients with NF1 and 4 patients with sporadic MPNST including febrile neutropenia (*n* = 6), anemia (*n* = 4), altered mental status, aphasia, and somnolence (*n* = 4) attributed to ifosfamide and secondary acute myeloid leukemia one year after completion of treatment in one patient with NF1 MPNST.

### 3.4. Central Pathology Evaluation

Diagnostic biopsies and surgical tumor specimens obtained on this trial were sent for centralized pathology review. Thirty-seven samples (26 NF1, 11 sporadic) were obtained prior to therapy and 9 (6 NF1, 3 sporadic) were obtained during treatment. Consistent with the diagnosis of high-grade MPNST, the majority of NF1 and sporadic MPNST had high cellularity, conventional histology, high mitotic rate, and presence of necrosis; p53 staining was positive in most tumors, and the proliferation index (MIB-1) was high in most tumors (for details, see Supplementary Appendix Table S4). There were no differences for responders or nonresponders (patients with disease progression) based on pathology features for NF1 and sporadic MPNST. In those patients who were considered evaluable after 4 cycles and had initial tissue available for FISH testing, the* TOPO2A* gene was amplified in 5 of 20 samples (NF1 *n* = 3, sporadic *n* = 2). Only one of these patients had partial tumor response, 3 patients had stable disease, and one NF1 patient had progressive disease.

## 4. Discussion

The overall survival of unresectable or metastatic MPNST remains poor and thus there is a critical need for the development of effective medical treatments. This was the first trial to prospectively evaluate the objective response rate in high-grade chemotherapy-naive MPNST using an identical chemotherapy regimen and stratification for NF1 and sporadic MPNST. Responses were determined separately for sporadic and NF1-associated MPNST, as response to chemotherapy and outcome have been reported to be worse for NF1-associated compared to sporadic MPNST as noted by Ferrari et al. [3, 14]. Similar to these studies, our study also observed a lower response rate for NF1 MPNST (17.9%) compared to sporadic MPNST (44.4%).

However, our study was not powered to detect a difference in objective response rates between the two strata. This trial enrolled patients slowly over 6 years, and we closed the study to enrollment when it was clear that enrollment could not be completed within an acceptable time frame. With 5/28 partial responses in NF1 MPNST, it is unlikely that the desired 40% OR rate (≥11/37 patients) would have been met, even with completed accrual. This trial may have recruited slowly, as the treatment consisted of conventional chemotherapy drugs; that is, the agents were not novel and are available at most sites where they are used for the treatment for sarcomas. While the desired response rates were not achieved, the chemotherapy regimen selected clearly resulted in disease stabilization and minor decreases in the target lesions were observed in most patients. This is in contrast to recently completed clinical trials with targeted agents, in which most patients have experienced rapid disease progression [32–36]. IA resulted in disease stabilization and minor decreases in tumor size in most patients, with additional shrinkage observed following IE in several patients. The inclusions of etoposide for MPNST differ from the most frequently used regimen of ifosfamide and doxorubicin. Consideration should be given to etoposide in conventional chemotherapy regimens for patients with MPNST as five patients in this study had shrinkage after two cycles of IE despite growth with IA.

Disease stabilization and small reductions in tumor size in addition to the partial responses were seen in patients with NF1 and sporadic MPNST and appeared similar in magnitude (Figure 2). Thus, while the partial response rate was lower in NF1 compared to sporadic MPNST, qualitatively, responses were similar. This suggests that there may be a role for chemotherapy in NF1 and sporadic MPNST and that the combination of chemotherapy with targeted agents may be one strategy to identify more effective agents for MPNST.

A study using cDNA microarray analysis found that* TOPO2A* was the most overexpressed gene in MPNST compared with benign neurofibromas. Excess copies of* TOPO2A* were also seen at the DNA level in 10 of 16 cases, while increased expression of TOPO2A protein was seen in 83% of the tumors on a tissue microarray. The TOPO2A-expressing tumors were also associated with poor cancer-specific survival and presence of metastases [29]. Topoisomerase II is the primary target for several anticancer agents including doxorubicin and etoposide; the hope was that the MPNST being more likely to express* TOPO2A* would be more sensitive to these chemotherapy agents.* TOPO2A* was assessed by FISH and only 5/20 response-evaluable MPNST were found to harbor gene amplification. In addition, only one of these patients had a partial response to the treatment; however, this is too small of a sample size to draw firm conclusions. Additional pathologic analyses did not shed light on additional markers of response. This study allowed for clinical evaluation of patients with NF1 manifestations at trial enrollment, which has not been done in previous trials. In general, in the adult NF population the percentage of patients with ≥10 subcutaneous neurofibromas ranges between 14 and 23% while in our study it was 41% [37]. Presence of subcutaneous neurofibroma burden has been associated with a greater risk for MPNST in NF1 in the past [38, 39].

Our trial compared response assessment by WHO and RECIST which was mostly in agreement. We selected WHO over RECIST as many MPNST are not spherical. We recommend additional comparison in future clinical trials for MPNST to evaluate which method is preferable. Another goal of our study was to assess the utility of FDG-PET in assessing the response to therapy. However only 25 of 48 patients had initial PET scans and only 13 patients had follow-up PET after cycle 5 which did not allow for a meaningful analysis.

In summary, similar to previous trials, our prospective study described a lower objective response rate in NF1-associated compared to sporadic MPNST. While our study did not meet final accrual and was not powered to detect differences in response rates between sporadic and NF1-associated MPNST, this chemotherapy regimen used resulted in disease stabilization in a majority of the patients, which is important in a highly aggressive tumor. Novel treatment strategies for MPNST, including evaluation of the combination of cytotoxic and targeted therapies, should be considered.

## Supplementary Material

Table S1: Chemotherapy Schema. Table S2: On Study Evaluation. Table S3: Evaluation of patients based on response after cycle 4. Table S4: Central pathology evaluation of tumor samples prior to treatment.

## Figures and Tables

**Table 1:** Patient characteristics at enrollment and response evaluation.

Stratum	NF1 MPNST	Sporadic MPNST
Eligible patients enrolled	34	14

Male/female	22/12	9/5

Median age: years (range)	33 (8–66)	40 (13–72)

Race: *n* (%)		
White, non-Hispanic	14 (41%)	11 (79%)
Black	13 (38%)	2 (14%)
Hispanic	4 (12%)	0 (0%)
Other/unknown	3 (9%)	1 (7%)

ECOG score: 0/1/2	4/24/6	6/5/3

Disease: localized/metastatic	18/16	5/9

Location		
Head	2	0
Neck	3	1
Chest	10	4
Abdomen/pelvis	9	5
Spine	5	1
Upper extremity	3	1
Lower extremity	2	2

Response evaluation after cycle 4	28	9

Complete response (CR)	—	—
Partial response (PR)	5	4
Stable disease (SD)	20	4
Progressive disease (PD)	3	1

Objective response rate: %	17.9	44.4

**Table 2:** Baseline NF1 clinical findings in 34 patients with NF1 MPNST.

*NF1 diagnostic signs (number of subjects with data provided)*	
6 or more CAL (*n* = 28)	17
Intertriginous freckling (*n* = 28)	19
Neurofibromas (*n* = 28)	26
Subcutaneous neurofibromas (*n* = 24) (0/1–9/≥10)	4/10/10
Cutaneous neurofibromas (*n* = 24) (0/1–9/≥10)	6/5/13
Plexiform neurofibroma (*n* = 20)	12
Paraspinal neurofibromas (*n* = 24)	12
Glioma/optic glioma (*n* = 18)	1/1
*Other morbidity*	
Scoliosis (*n* = 22)	5
Intellectual delay (*n* = 26)	8
Hypertension (*n* = 28)	8
