# Supplementary material for: SARC006: Phase II Trial of Chemotherapy in Sporadic and Neurofibromatosis Type 1 Associated Chemotherapy-Naive Malignant Peripheral Nerve Sheath Tumors
Source: Sarcoma. 2017 Sep 12;2017:8685638. doi: 10.1155/2017/8685638 (PMC5613633; doi:10.1155/2017/8685638)
Supplement: Supplementary file 1 — Table S1: Chemotherapy Schema. Table S2: On Study Evaluation. Table S3: Evaluation of patients based on response after cycle 4. Table S4: Central pathology evaluation of tumor samples prior to treatment. [file 8685638.f1.pdf]

SUPPLEMENTARY APPENDIX

Table S1: Chemotherapy Schema

Patients receiving radiation therapy for local control

Cycle	1	2	3	4	5	6	7	8
Week	0	3	6	9	12	15	18	21
Drugs	Ifos<br>Doxo<br>Dxrz*<br>Mesna<br>G-CSF/<br>Neulasta	Ifos<br>Doxo<br>Dxrz<br>Mesna<br>G-CSF/<br>Neulasta	Ifos<br>Etop<br>Mesna<br>G-CSF/<br>Neulasta	Ifos<br>Etop<br>Mesna<br>G-CSF/<br>Neulasta	Ifos<br>Etop<br>Mesna<br>G-CSF/<br>Neulasta	Ifos<br>Etop<br>Mesna<br>G-CSF/<br>Neulasta	Ifos<br>Doxo<br>Dxrz<br>Mesna<br>G-CSF/<br>Neulasta	Ifos<br>Doxo<br>Dxrz<br>Mesna<br>G-CSF/<br>Neulasta

Patients not receiving radiation therapy for local control

Cycle	1	2	3	4	5	6	7	8
Week	0	3	6	9	12	15	18	21
Drugs	Ifos<br>Doxo<br>Dxrz<br>Mesna<br>G-CSF/<br>Neulasta	Ifos<br>Doxo<br>Dxrz<br>Mesna<br>G-CSF/<br>Neulasta	Ifos<br>Etop<br>Mesna<br>G-CSF/<br>Neulasta	Ifos<br>Etop<br>Mesna<br>G-CSF/<br>Neulasta	Ifos<br>Doxo<br>Dxrz<br>Mesna<br>G-CSF/<br>Neulasta	Ifos<br>Doxo<br>Dxrz<br>Mesna<br>G-CSF/<br>Neulasta	Ifos<br>Etop<br>Mesna<br>G-CSF/<br>Neulasta	Ifos<br>Etop<br>Mesna<br>G-CSF/<br>Neulasta

Dxrz =Dexrazoxane 375 mg/ m^2/ dose IV over 15-30 minutes immediately prior to Doxo on days 1, 2

*Dexrazoxane use was not mandatory and was administered per institutional guidelines.

Doxo =Doxorubicin 37.5 mg/ m^2/ dose IV over 15 minutes on days 1, 2

Ifos =Ifosfamide 1,800 mg/ m^2/ dose IV over 60 minutes on days 1, 2, 3, 4, 5

Etop = Etoposide 100 mg/ m^2/ dose IV over 60 minutes on days 1, 2, 3, 4, 5

Mesna =Mesna 360 mg/ m^2/ dose IV, first dose over 60 minutes (with Ifos) and doses 2-8 over 15 minutes every 3 hours on days 1, 2, 3, 4, 5 with Ifos. Other standard mesna dosing regimens including oral administration of mesna (360 mg/ m2 PO q 3hr supplied as scored 400mg tablets), used at participating institutions, were also permissible.

G-CSF = Filgrastim 5µg/kg/dose SC daily starting 24-36 hours after last dose of chemotherapy until post-nadir ANC ≥1,500/µl, or peg-filgrastim (neulasta™) for patients weighing ≥ 45kg: 6mg SC x 1 dose starting 24 hours after completion of chemotherapy. Institutional guidelines could be used for hydration and preparation and administration of the standard chemotherapy and supportive care agents

Table S2: On Study Evaluation

	Prior to therapy	Prior to cycles	During treatment cycles
History and Physical exam	X	All Cycles	
Phenotypic analysis	X		
CBC, differential	X	All cycles	Weekly to twice weekly[@]
PT,PTT, fibrinogen	X		
Electrolytes[§], Ca, Mg, Phos	X	All cycles	Weekly
Liver function tests[¶]	X	All cycles	Weekly
Urine pregnancy test	X	3,5,7	
MUGA or ECHo	X	Pre cycle 3 doxo	
Chest X-ray	X	As clinically indicated	
Plain film of primary disease	X	As clinically indicated	
CT of chest	X	Cycles 3,5,7	
MRI of primary	X	Cycles 3,5,7	
^{18}FDG-PET	X	Cycle 5	
Cr Cl*	X		
Urinalysis	X	All cycles [¥]	
Serum proteomics	X	Cycles 5**, 7	
Tumor specimen	X	Cycle 5 (if surgery for local control)	

@ Monitor CBC twice weekly once ANC ≤1,000/μL until ANC recovers to ≥500/μL

§ Na, K, CL, CO_2, BUN, creatinine, glucose

¶ ALT, AST, bilirubin, alkaline phosphatase, LDH

* IF serum creatinine abnormal

¥ Frequency of urinalysis during ifosfamide treatment will follow institutional guidelines

**After recovery from cycle 4

Table S3. Evaluation of patients based on response after cycle 4

Stratum	NF1 MPNST	Sporadic MPNST
Response evaluation post cycle 4	28	9
Complete Response (CR)	-	-
Partial Response (PR)	5	4
Stable Disease (SD)	20	4
Progressive Disease (PD)	3	1
Initial tumor size > 5cm*		
PR	3	3
SD	19	3
PD	3	1
Initial tumor size < 5cm*		
PR	2	1
SD	1	1
PD	0	0
Metastatic disease at diagnosis (stage 4)		
PR	1	2
SD	10	2
PD	0	0
Non-metastatic disease at diagnosis (stage 3)		
PR	4	2
SD	10	2
PD	3	1
≥33 years old		
PR	3	4

SD	10	2
PD	1	1

*largest diameter

Table S4: Central pathology evaluation of tumor samples prior to treatment

		NF1 (n= 26)	Sporadic (n= 11)
Location	Central	6	2
	Nerve plexus	3	0
	Trunk	10	6
	Head and Neck	2	0
	Retroperitoneal	1	0
	Proximal limb	2	1
	Distal limb	2	1
	Metastasis	0	1
Arising from Neurofibroma	Yes	13	0
	No	5	9
	Unclear	8	2
Histologic Variant	Conventional	15	7
	Perineural	1	0
	Epithelioid	0	2
	Divergent (Triton)	1	0
	Mixed histology	9	2
Extracellular mucin	0	14	6
	1	9	3
	2	3	1
	3	0	1
Intravascular invasion		7	2

Cellularity	low<br>moderate<br>high	0<br>5<br>21	0<br>4<br>7
Cellular pleomorphism	1<br>2<br>3	9<br>14<br>3	4<br>5<br>2
Nuclear atypia	1<br>2<br>3	4<br>17<br>5	1<br>5<br>5
Nuclear chromatin	bland<br>hyperchromatic<br>vesicular<br>hyperchromatic/vesicular	1<br>19<br>3<br>3	2<br>3<br>6<br>0
Macronucleoli		8	5
Max #mitoses per 10x HPF		44	44
Atypical mitoses		4	4
Small cells (high NC ratio)	absent<br>1-10%<br>10-50%	20<br>1<br>4	7<br>1<br>3
Necrosis	absent<br>1-10%<br>10-50%<br>>50%	5<br>8<br>10<br>3	1<br>2<br>5<br>3

Nuclear pseudopalisading		6	3
**IMMUNOHISTOCHEMISTRY**			
s100		17+ (of 23)	8+ (of 11)
Leu-7		12+ (of 23)	4 (of 11)
EMA		3 (of 21)	3 (of 11)
Desmin		6 (of 23)	0 (of 11)
p53		16 (of 22)	10(of 11)
Ki67 (MIB-1)		Mean: 31%	Mean: 50%
EGFR		19 (of 21)	8 (of 11)
**FISH**			
EGFR (7p12) amplification		3 (of 20)	1 (of 8)
TOPO2A (17q21-q22) amplification		5 (of 19)	2 (of 10)
Her2/Neu (17q11-q12) amplification		2 (of 18)	0 (of 10)
Cyclin D1 (11q13) amplification		1 (of 19)	2 (of 9)
c-MYC (8q24) amplification		5 (of 20)	0 (of 8)
N-MYC (2p24) amplification		3 (of 16)	1 (of 6)
NF1 (17q11) deletion		7 (of 19)	0 (of 9)

p16 (9p21) deletion		10 (of 14)	3 (of 6)
RB (13q14) deletion		1 (of 17)	2 (of 10)
p53 (17q13) deletion		6 (of 19)	2 (of 9)
